# Cancer: A Hundred Years Ago and To-Day

**Published:** 1889-02-23

**Authors:** 


### WITHIN THE WARDS.

CANCER: A HUNDRED YEARS AGO AND TO-DAY. To men, and still more to women, cancer is a subject of deep, and often of painful interest. Memory recalls those who have first endured, and then died of its ravages: fear looks forward to a time when in spite of care the most anxious, and precaution the most watchful, the dread disease may attack ourselves or those we love better than ourselves. How stands medical science to-day in relation to the destroyer? Is it where it was a hundred years ago, or has any advance been made? It would not be true to say that such a degreee of progress has been made as readily appeals to the untrained mind of the outsider. Nevertheless, it is certain that even in regard to the treatment and cure of cancer medical science occupies a decidedly higher platform than it did a hundred years ago. One of the most illustrious names on the roll of medical fame is that of John Hunter. A hundred years ago he represented all that was profoundest, broadest, most philosophic, and most practical in the world of medicine. On the subject of "cancer," John Hunter then wrote: "No cure has yet been found; for what I call a cure is an alteration of the disposition and the effect of that disposition, and not the destruction of the cancerous parts. But as we have no such medicine we are often obliged to remove cancerous parts, which extirpation, however, will often cure as well as we could do by changing the disposition and action. Arsenic seems to have some power of this kind, and its effects might be increased by being used internally and externally; but its use is very dangerous, and, I am afraid, insufficient for the disease. This is a remedy which enters into the empirical nostrums which are in vogue for curing cancer, and among which Plunkett's holds the highest rank. But this is no new discovery, for Senertus, who lived the Lord knows (*sic*) how long ago, mentions a Roderiguez and Flusius, who obtained considerable fame and fortune by such a composition. I was desired to meet Mr. Plunkett, to decide on the propriety of using his medicine in a particular case. I have no objection to meet anybody. It was the young one (Plunkett); the old one is dead, and might have died himself of a cancer for aught I know. I asked him what he intended to do with his medicine. He said, 'To cure the patient.' 'Let me know what you mean by that. Do you mean to alter the diseased state of the parts, or do you mean by your medicine to remove the parts diseased?' 'I mean to destroy them,' he replied. 'Well, then, that is nothing more than either I or any other surgeon could do, with less pain to the patient.' Poor Woollett, the engraver, died under one of these cancer curers. He was under my care when this person took him in hand. He (the cancer-curer) had been a life-guardsman, I think, and had got a never-failing receipt. I continued to call upon Woollett as a friend, and received great accounts of the good effects, upon hearing which I said, if the man would allow me to watch regularly the appearance of the cancer, and see myself the good effects, and I should be satisfied of its curing only that cancer (mind, not by *destroying* it), I would exert all my power to make him the richest man in the kingdom. But he would have nothing to do with me, and tortured poor Woollett for some time, till at last I heard the sound parts were gone, and at length he died."

This extract from John Hunter's writings demonstrates accurately the state of knowledge and judgment within the medical profession a hundred years ago. It also shows what was the condition of ignorance and pretence outside. By a similar extract from a modern writer of eminence it is possible to make clear the state of knowledge and opinion in the highest professional circles on the subject of cancer to-day. It is well known that the removal of a new growth at a very early stage will frequently effect a "cure," and that of a permanent character, even though the growth, if left to itself, might ultimately have shewn cancerous characteristics. But concerning cases which are so far advanced as to leave doubt of their real nature, or of recurring "cancer" growths what has the surgical science of the present time to say? Sir Spencer Wells, than whom modern surgery has no more distinguished professor, says: "We may be consulted in cases where the period for early and hopeful operation has passed by, or where months or years after operation, recurrence has taken place—it may be that recurring growths have also been removed, once or several times—and then comes a time when a patient, exhausted by loss of blood or by profuse offensive discharge, approaches the end, and further operative treatment is abandoned. Are we to be content with relieving pain, prolonging sleep, and with the use of deodorising or disinfecting appliances? Or can we do more? Can we encourage the folorn hope of the patient that even yet growth may be stopped; that some benign alteration may take place in its structure—something like the spontaneous involution of Virchow—some retrogade fatty change in the cells or elementary components of the tumour, while extension or growth of new cancer cells is stopped? If this could be attained before the whole body were infected, it would amount to curing cancer. From time to time such cases of cure are reported, but few, if any, stand the test of accurate investigation. Virchow, however, cautions us against too great scepticism in such therapeutical investigations, and says that the hopeless condition of the patient justifies the trial of remedies of whose mode of action we have no clear idea. Whether we can go still further and secure for healthy tissues immunity against infection from an existing cancer--a resistance to invasion—remains a subjects for future investigation. All we can yet say is that under certain conditions the nutrition of cancer cells may be so far arrested as to lead to their complete disintegration. This is often observed in small fragments, or within limited circumferences, and Nussbaum has recently succeeded in the attempt to effect such changes in large growths by cutting off their supply of blood. He makes deep furrows with Paquelin's cautery through the integuments and subcutaneous fat quite down to the adjacent fascia or muscles, thus cuts off all peripheral supply of blood, and so lessens the abnormal vascularity of many malignant tumours. Soft fungoid masses which bleed readily soon become firm, and the whole tumour more solid, while general health improves and life is prolonged. And we also hear from Munich of aims at a somewhat similar object by injecting solutions of ozone into the substance of cancerous growths and the tissues surrounding them. Not only is the proliferation of epithelial cells said to be arrested, and the formation of cicatrical tissue obtained, but the surrounding textures are protected against invasion. So long as the disease is local it must be curable. As Virchow says, 'If cancer in its beginning, and often very long afterwards, is a local disease, it must be possible during this period locally to cure it.'" The advanced position which surgical science holds to-day as against that held a hundred years ago consists in the growing conviction that at the outset cancer is a local disease; that thoroughly scientific, rational, and successful methods of dealing with local diseases are now in general use; that all the world over the ablest minds in medicine are directed with even painful intensity to the practical study of the subject, and that every reasonable medical man agrees with Virchow that the "condition of the patient justifies the trial of remedies of whose mode of action we have no clear idea." It will be strange indeed if, with advancing knowledge and the gradual emancipation of the medical mind from prejudice and routine, there be not found some simple, but effective method of checking the ravages of this fatal disease.

